# Marine crude-oil biodegradation: a central role for interspecies interactions

**DOI:** 10.1186/2046-9063-8-10

**Published:** 2012-05-16

**Authors:** Terry J McGenity, Benjamin D Folwell, Boyd A McKew, Gbemisola O Sanni

**Affiliations:** 1 School of Biological Sciences, University of Essex, Wivenhoe Park, Colchester, CO4 3SQ, UK

**Keywords:** Hydrocarbon, Crude oil, Salt marsh, Marine microbiology, Biodegradation, Bioremediation, Microbial interactions, Biogeochemistry, *Alcanivorax*

## Abstract

The marine environment is highly susceptible to pollution by petroleum, and so it is important to understand how microorganisms degrade hydrocarbons, and thereby mitigate ecosystem damage. Our understanding about the ecology, physiology, biochemistry and genetics of oil-degrading bacteria and fungi has increased greatly in recent decades; however, individual populations of microbes do not function alone in nature. The diverse array of hydrocarbons present in crude oil requires resource partitioning by microbial populations, and microbial modification of oil components and the surrounding environment will lead to temporal succession. But even when just one type of hydrocarbon is present, a network of direct and indirect interactions within and between species is observed. In this review we consider competition for resources, but focus on some of the key cooperative interactions: consumption of metabolites, biosurfactant production, provision of oxygen and fixed nitrogen. The emphasis is largely on aerobic processes, and especially interactions between bacteria, fungi and microalgae. The self-construction of a functioning community is central to microbial success, and learning how such “microbial modules” interact will be pivotal to enhancing biotechnological processes, including the bioremediation of hydrocarbons.

## The problem of marine oil pollution

**Figure 1:**
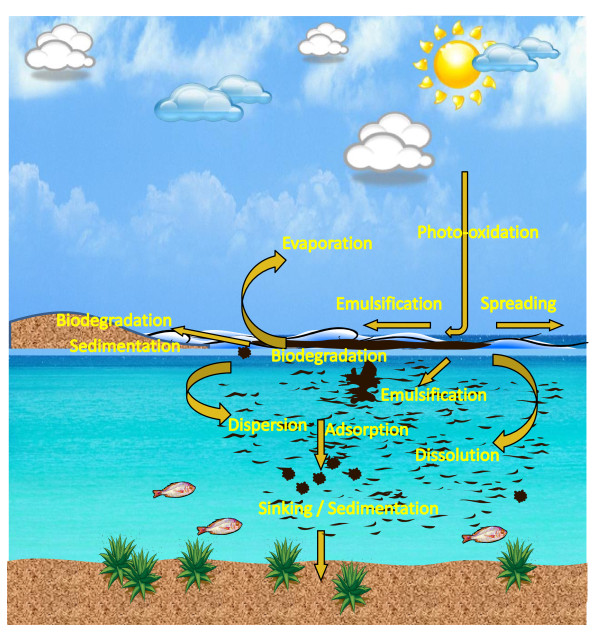
**Fate of a marine oil spill (for a more detailed explanation, see**http://www.itopf.com/marine-spills/fate/weathering-process/). Spreading is affected by the action of winds, waves, water currents, oil type and temperature, and enhances evaporation of the volatile fractions such as low molecular weight alkanes and monoaromatic hydrocarbons. Spilt oil is broken into droplets and dispersed through the water column, enhancing the biodegradation of hydrocarbons and dissolution of water-soluble fractions of oil. Turbulent seas cause water droplets to be suspended in the oil, resulting in water-in-oil emulsions, alternatively known as chocolate mousse, which is difficult to degrade because of its high viscosity and reduced surface area. Photo-oxidation is the process by which hydrocarbons, especially PAHs, react with oxygen in the presence of sunlight, resulting in structural changes that can on the one hand lead to increased water solubility or, conversely, increased recalcitrance to biodegradation. Sedimentation will general only occur when oil adsorbs to particles owing to nearly all crude oils having a lower density than seawater.

Our seas, oceans and coastal zones are under great stress; and pollution, particularly by crude oil, remains a major threat to the sustainability of planet Earth [1]. An estimated 1.3 million tonnes of petroleum enters the marine environment each year [2]. Acute pollution incidents cause great public concern, notably ~600,000 tonnes of crude oil released after the Deepwater Horizon explosion in the Gulf of Mexico [3] and ~63,000 tonnes from the Prestige oil-tanker [4] off the coast of north-west Spain. The fate of crude oil spilled at sea (Figure 1) depends on both the prevailing weather and the composition of the oil; but its environmental impact is exacerbated on reaching the shoreline, especially in low-energy habitats, such as lagoons and salt marshes. Acute pollution events can result in mass mortality; for example, more than 66% of total species richness (including polychaetes, molluscs, crustaceans and insects) was lost in the worst affected beaches following the Prestige spill [5]. Hydrocarbons also contaminate the feathers and fur of marine birds and mammals, resulting in the loss of hydrophobic properties, leading to death from hypothermia [6], or lethal doses following ingestion of oil during preening. 

Moreover, the impact of hydrocarbons, especially polycyclic aromatic hydrocarbons (PAHs), on wildlife and fisheries may be long-lasting; for example the Fisheries Exclusion Zone imposed after the Braer spill (Shetland Islands, United Kingdom, 1993) due to contaminated fish and shellfish, remained in place for over 6 years. Chronic pollution can cause physiological or behavioural damage at sub-lethal concentrations; and genetic damage and decreases in both growth and fecundity have been observed in fish [7,8]. Deep-sea sediments and associated biota are also chronically affected by drilling, which deposits vast amounts of oil-contaminated drill cuttings on the seafloor [9]. Even when oil-contaminated coastal sediments appear to be clean (e.g. Prince William Sound that was contaminated by the Exxon Valdez spill in 1989), toxic oil components, such as high molecular weight (HMW) PAHs, may remain buried and sorbed to sediment particles, and can be released to the environment by bioturbation or human activities such as dredging [10].

**Figure 2:**
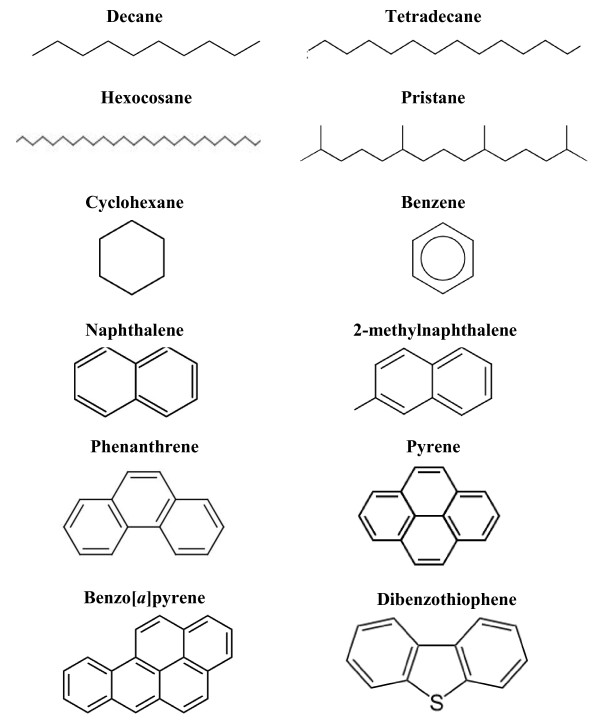
Structure of selected components of petroleum.

Crude oil is a natural, heterogeneous mixture of hydrocarbons, with potentially 20,000 chemical components [11], consisting mainly of alkanes with different chain lengths and branch points, cycloalkanes, mono-aromatic and polycyclic aromatic hydrocarbons (Figure 2; [12]). Some compounds contain nitrogen, sulfur and oxygen [12]; while trace amounts of phosphorus, and heavy metals such as nickel and vanadium are also found [13]. Its composition varies widely, and each oil component has different physico-chemical properties, including viscosity, solubility and capacity to absorb (Table 1), as well as varying in its bioavailability and toxicity. Crude oil, released naturally from the geosphere to the biosphere (e.g. from cold seeps [14]) may supply up to half of the oil in the sea [2]. Although hydrocarbons are relatively stable molecules, their “fuel value” and presence in the environment for millions of years have led to the evolution of many microbes able to activate and use them as a major or sole source of carbon and energy, including at least 175 genera of Bacteria [15]. Several haloarchaeal genera [16] and many Eukarya can grow on or transform hydrocarbons [17]. Biodegradation of crude oil to carbon dioxide and water is the major process by which hydrocarbon-contaminated environments are remediated. 

**Table 1:** Selected hydrocarbons and their solubility in deionised water at 25°C and hydrophobicity indicated as Log K_ow_

**Compound**	**Solubility (mg L**^**-1**^**)**	**Log K**_**ow**_
Decane	0.091	6.1
Tetradecane	0.009	7.2
Hexocosane	NA	14.7
Pristane	5 × 10^-5^	11.4
Cyclohexane	43.0	3.2
Dibenzothiophene	2.41	4.3
Benzene	1790	2.1
Naphthalene	31.7	3.3
2-methylnaphthalene	24.6	3.9
Phenanthrene	1.29	4.5
Pyrene	0.14	5.3
Benzo[*a*]pyrene	0.004	6.0

## The principal marine hydrocarbon degraders

The starting point in elucidating potential complex interactions involved in hydrocarbon biodegradation is to identify the microbes primarily responsible for biodegradation, and their catabolic pathways. It has long been known that the enzymatic activation of hydrocarbons by oxygen is a pivotal step in their biodegradation, and several mechanisms have been elucidated for aromatic [12,18,19] and aliphatic [12,20] compounds. However, our understanding of the catabolic processes for HMW PAHs [21] and anaerobic activation mechanisms and pathways, e.g. fumarate addition, carboxylation and O_2_-independent hydroxylation, have emerged only recently [22-25].

The microbial response to an oil spill at sea is dependent on numerous factors, including the oil composition and degree of weathering, as well as environmental conditions, particularly temperature and nutrient concentrations. Nevertheless, there are some typical patterns; most notable is the large increase in abundance of *Alcanivorax* spp., which degrade straight-chain and branched alkanes [26-32], followed by *Cycloclasticus* spp., which degrade PAHs [26-30,33-36].

Since the cultivation of *Alcanivorax borkumensis*[37], functional genomic, biochemical and physiological analyses have revealed the underlying basis of its success [28,38-40]. While it lacks catabolic versatility, utilising alkanes almost exclusively as carbon and energy sources, it has multiple alkane-catabolism pathways, with key enzymes including alkane hydoxylases (a non-haem diiron monooxygenase; AlkB1 and AlkB2) and three cytochrome P450-dependent alkane monooxygenases [38]. Their relative expression is influenced by the type of alkane supplied as carbon and energy source and phase of growth [38]. *Alcanivorax borkumensis* also possesses a multitude of other adaptations to access oil (e.g. synthesis of emulsifiers and biofilm formation [38]) and to survive in open marine environments (e.g. scavenging nutrients and resistance to ultraviolet light [38,40]). *Acinetobacter* spp., which are commonly isolated from oil-contaminated marine environments [41], also have a diverse array of alkane hydroxylase systems enabling them to metabolize both short- and long-chain alkanes [20,42]. For example, *Acinetobacter* strain DSM 17874 contains a flavin-binding monooxygenase, AlmA, which allows it to utilize C_32_ and C_36_*n*-alkanes [43]. The *almA* gene has also been found in *Alcanivorax dieselolei* B-5 and is induced by long-chain *n*-alkanes of C_22_ - C_36_[44]. A diverse array of *alk*B gene sequences, encoding alkane hydroxylase, has been detected in the environment [45,46] and in a wide range of bacteria [38,42,46], however Païssé *et al*. [47] argue that *alk*B expression may not always be a good indicator for microbial oil degradation, implying that we have not fully explored the gene diversity and/or that other hydrocarbon catabolic processes were prevalent in the environment under investigation.

In cold marine environments, the obligate alkane-degrading psychrophile, *Oleispira*, rather than *Alcanivorax* spp., are commonly associated with oil spills [29,48]; and *Alcanivorax* spp. are sometimes outcompeted by *Thalassolituus* spp. in temperate environments [34]. Such obligate hydrocarbon-degrading bacteria can constitute 90% of the microbial community in the vicinity of the oil spill and have a wide global distribution [28]. New genera of obligate alkane degraders are still being discovered, e.g. *Oleibacter* sp. [31,49], and there are likely to be many more, such as the uncharacterised Oceanospirillales strain ME113 [50], which has been detected in abundance in other oil-rich marine environments [51,52].

The role of the generalists that degrade alkanes and/or PAHs as well as non-hydrocarbons is often overlooked, yet they can constitute a significant proportion of a hydrocarbon-degrading community. For example, Buchanan and Gonzalez [53] outline eight studies in which members of the *Roseobacter* lineage, which harbours a diversity of ring-hydroxylating dioxygenases and alkane hydroxylases, increase in abundance in hydrocarbon-enriched marine waters. Other generalists, including *AcinetobacterMarinobacter, Pseudomonas* and *Rhodococcus* spp. [54-57], contribute to hydrocarbon degradation. Sediments add to the complexity of identifying the main hydrocarbonoclastic microbes, but nearly all of the above genera are detected in the aerobic zone of marine sediments and presumed to be active in hydrocarbon degradation. It is important to recognise that within most of the genera labelled here as generalists (e.g. *Marinobacter*) there are many species, ranging from those that do not degrade hydrocarbons to specialists like *Marinobacter hydrocarbonoclasticus*, which almost exclusively utilises * n*-alkanes [56].

Although *Cycloclasticus* is frequently the main marine PAH-degrading microbe detected, many others from several tens of genera are known [15], and the underlying mechanisms of their interactions with, and degradation of PAHs are only beginning to be elucidated. For example, in San Diego Bay sediments, isolates able to grow on phenanthrene or chrysene were from the genera *VibrioMarinobacterCycloclasticusPseudoalteromonasMarinomonas* and *Halomonas*[58]. Another marine specialist PAH degrader, named *Porticoccus hydrocarbonoclasticus*, was recently isolated [59], and strains of *Microbacterium* and *Porphyrobacter*, previously not known to be involved in PAH degradation, were isolated on benzo*a*pyrene after enriching for two years [60]. Based on DGGE analysis, Hilyard *et al*. [61] suggested that Planctomyces and Bacteroidetes were involved in PAH degradation, and many more species from diverse genera that are implicated in PAH degradation remain to be cultivated, particularly those growing on HMW PAHs.

Incubation of marine sediment in the presence of phenanthrene and bromodeoxyuridine (BDU), followed by analysis of BDU-labelled DNA, revealed a remarkable diversity of putative PAH degraders belonging to the genera *ExiguobacteriumShewanellaMethylomonasPseudomonasBacteroides*, as well as Deltaproteobacteria and Gammaproteobacteria that were not closely related to cultivated organisms [62]. Some were also cultivated, including a novel *Exiguobacterium* strain, but the rest remain to be grown [62]. Similarly, stable-isotope probing (SIP) of DNA was used to identify the involvement of a novel clade of Rhodobacteraceae in biodegradation of low molecular weight (LMW) PAHs in marine algal blooms [63]. Obtaining pure cultures of the main microbes responsible for hydrocarbon biodegradation is no longer a prerequisite for their study, but it makes their investigation very much easier, allowing genomic, biochemical and physiological analyses that in turn can help to explain their *in-situ* function and interactions. It is also frequently their reliance on other microbes that prevents cultivation in the first instance, and growth in the proximity of microbes (or their diffusible products) from the same habitat [64] can be employed to improve recovery. Numerous other procedures can enhance cultivation [65], especially by increasing the bioavailability of hydrocarbons. Calvo *et al*. [66], for example, extracted extracellular polymeric substances (EPS) from *Halomonas eurihalina*, not a PAH-degrader, which enhanced the isolation of other microbes growing on PAHs.

## General considerations of microbial interactions

A volume of 1 mm^3^ of surface seawater, approximately equivalent to the size of a poppy-seed, contains ~600 bacteria, 150 cyanobacteria, 9 small algae, <1 protozoan [67] and ~10,000 viruses [68]. Numerous ecophsyiological investigations [69] together with modelling the co-occurrence of bacterial phylotypes [70] reveal a network of direct and indirect interactions within and between species in seawater that are vital for maintaining the microbial loop that drives marine biogeochemical cycles [71]. Some interactions exist between spatially separated species that use soluble or volatile metabolites to transmit information; while other interactions involve species in very close proximity, either as a biofilm on the same particle or physically associated to one another. Grossart [69] noted that a chain of the marine diatom, *Thalassiosira rotula*, can host up to 10^8^ bacteria [72], while a single copepod can harbour up to 10^9^ bacteria [73]. Surprisingly, in many studies, the attached microbiota, which is numerically equivalent to the non-attached microbiota, is removed by pre-filtration [69], and so not considered.

Microbial communities from coastal sediments vary more from one location to another than those from open waters, and have much greater community evenness [74]. Moreover, in sediments, cells are much more concentrated, resulting in a greater likelihood of interactions, which becomes even more prevalent in biofilms where cells are more densely packed. Highly productive photosynthetic microbial mats develop at the water-sediment interface. These multispecies biofilms consist of horizontally stratified layers with extremely steep gradients of light, redox potential, oxygen, sulfur species etc. The exceptionally high microbial diversity within a few microns covers a large range of metabolic groups (oxygenic and anoxygenic phototrophs, sulfate reducers, methanogens etc.) [75]. We are at an early stage in our understanding of communication mechanisms in each of these environments (open water, sediment and biofilms), where small molecules, either diffusing from cell to cell [76], or transported by vesicles [77] or via nanotubes bridging cells [78], elicit intra- and inter-species effects that could be antagonistic or beneficial.

Microbes exhibit all of the types of social behaviour (mutual benefit, selfishness, altruism and spite [79]) seen in multicellular organisms. However, it is often difficult to categorise such behaviour in complex multi-species natural environments, and so in this review we talk largely in terms of cooperation and competition, and how they are affected by hydrocarbons, and in turn influence their fate. Our knowledge gained from studying pure cultures of hydrocarbon degraders is important, but hydrocarbonoclastic bacteria rarely, if ever, function in isolation in nature. Therefore, a better understanding of crude-oil biodegradation, and thus the capability to more rationally remediate contaminated environments, requires us to consider the mechanisms of the interactions between different hydrocarbon-degrading microbes and with non-degrading organisms [27]. This review considers such interactions, with most emphasis on aerobic processes and interactions between phototrophic microalgae and hydrocarbonoclastic bacteria.

## Interactions between microbes during aerobic degradation of hydrocarbons

When crude oil is added to seawater, the microbial community changes and consists of multiple co-existing species [80], which can be explained most simply by resource sharing. As indicated above, crude oil consists of a variety of chemically distinct hydrocarbons, which require specific mechanisms for activation and degradation. In seawater microcosms, each supplied with a different hydrocarbon, McKew *et al*. [34] observed that: 1) *Alcanivorax* dominated when the branched alkane, pristane, was supplied, but was not detected in other microcosms, 2) *Cycloclasticus* was dominant with most PAHs, but was undetected when fluorene was supplied, and 3) *Thalassolituus* was the dominant species when *n*-alkanes with 12 to 32 carbons were added, but was not detected when decane was the sole alkane added to seawater. Thus, it appears that the ability to be competitive in the marine / estuarine environment requires that hydrocarbonoclastic bacteria are relatively specialised. Probably the extra genetic and cellular load needed to allow bacteria to grow on a wider range of hydrocarbons would demand greater nutrient resources, making them less competitive overall, especially in oligotrophic oceans. This, in turn, requires the presence of a consortium of microbes for complete degradation of crude oil.

Competition for resources is also an important element of petroleum biodegradation: all known *Alcanivorax* spp. can degrade *n*-alkanes, yet in the above study [34]*Thalassolituus* out-competed *Alcanivorax*. Furthermore, in a follow-up study *Alcanivorax* was undetected in the microcosms to which *Thalassolituus oleivorans* had been added previously, whereas it grew in all other microcosms, though its abundance was negatively correlated with that of *Thalassolituus*[30]. The nature of this competition deserves more detailed study. It could simply be competition for common resources, such as nutrients, but the idea that *Thalassolituus* actively releases bioactive compounds to inhibit competitors must be considered. Nevertheless, as noted above, in most oil-amended experiments and environmental surveys *Alcanivorax* is the dominant microbe, so it is pertinent to consider whether it produces antibacterial molecules. *Alcanivorax jadensis* produces an antibiotic which has been termed “alcanivorone” [81], but the impact of this antibiotic on other microorganisms during hydrocarbon degradation is still unknown. In a two-species experiment, *Alcanivorax borkumensis* outcompeted *Acinetobacter venetianus*, but the filtered spent medium from *Alcanivorax borkumensis* did not influence the growth of *Acinetobacter venetianus*, rather Hara *et al*. [82] proposed the former’s ability to use branched alkanes as a key factor. However, such branched alkanes are a relatively minor component of crude oil, and so the extra carbon and energy available to *Alcanivorax borkumensis* may be just one of several possible explanations.

Even when a single hydrocarbon is added to seawater microcosms, multiple species are always detected [34,36,80,83], and frequently mixed cultures outperform single species isolated from a consortium [83]. For example, the dominant benzo*a*pyrene-degrading bacteria from a marine enrichment were isolated, and faster degradation was seen when the three strains (*OchrabactrumStenotrophomonas* and *Pseudomonas* spp.) were combined than when tested individually [84]. Both *Cycloclasticus* and *Pseudomonas* were abundant in estuarine waters enriched with naphthalene, but *Pseudomonas* appeared in the latter stages of the enrichment [36]. Perhaps the most compelling explanation for multiple species growing on one carbon and energy source, is that a measurable amount of the PAH is not completely oxidized to CO_2_ and H_2_O by one organism, resulting in oxidation products being liberated into the environment. Numerous microbes may take advantage of this so-called epimetabolome [85,86] as sources of carbon and energy [87,88].

It is becoming apparent that metabolite sharing is widespread in nature and in the laboratory as shown using auxotrophic mutants of *Escherichia coli* that complemented each other's growth by cross-feeding essential metabolites [89]. The cooperative behaviour of microbes to self-construct a functioning community is central to their success, and learning how such “microbial modules” interact will be pivotal to enhancing biotechnological processes, including the bioremediation of hydrocarbons. However, few studies have tracked the flow of hydrocarbon-derived metabolites between microbes in a consortium, and many interesting metabolites are transient and therefore difficult to detect. Pelz *et al*. [87] tracked the biodegradation of 4-chlorosalicylate through a three-member consortium of *Pseudomonas* MT1, *Pseudomonas* MT4 and *Achromobacter* MT3 using ^13^C-labelled substrates. Analysis revealed a network of carbon sharing: strain MT1, the only member able to degrade 4-chlorosalicylate, provided carbon skeletons to the other strains (MT3 and MT4), while they degraded toxic metabolites that inhibited strain MT1 if allowed to accumulate [87]. One of the toxic intermediates (4-chlorocatechol) was partially taken up by strain MT3 and further degraded [87]; and a proteomic and metabolite analysis of a co-culture of strains MT1 and MT3 revealed the importance of strain MT3, not only in consuming the toxic intermediate but also in reducing the degradation rate of the parent compound by strain MT1; both of which minimized the stress experienced by strain MT1 as judged by negligible detection of stress-response proteins in the mixed culture compared with the pure culture [90].

Reducing the stress imposed by metabolites may also be a typical feature in bacterial members of consortia degrading PAHs. However, our current knowledge of the catabolic routes for PAH degradation requires considerable development as diverse novel metabolites are produced by PAH-degrading microbes [43,91]; for example *Cycloclasticus* strain P1, derived from a deep-sea pyrene-degrading consortium, produced three metabolites, two of which could be identified as cyclopenta*d,e*,f]phenanthreone and 4-phenanthrenol [83]. These metabolites are unusual as they involve the creation of a pentagonal ring suggesting a novel catabolic pathway is adopted by strain P1 [83].

Chen and Aitken [92] showed that salicylate, an intermediate produced by a *Pseudomonas* sp. pre-grown on phenanthrene as a sole source of carbon and energy, induced production of a PAH dioxygenase leading to degradation of HMW PAHs that the isolate could not use for growth [92]. The importance of metabolites as inducers of co-metabolic degradation may be significant also in natural communities.

A wide variety of fungi are known to be important in initiating biodegradation of HMW PAHs in terrestrial environments by co-metabolism using a battery of enzymes (e.g. lignin peroxidases, manganese peroxidases, laccases and epoxide hydrolases) that probably evolved to breakdown other compounds such as lignin, but which fortuitously degrade PAHs [91,93-95]. Extracellular enzymes and radicals produced by ligninolytic fungi are not constrained by slow desorption and mass transfer which limit the activity of those microbes that need PAHs to enter the cell. Moreover, these metabolites are generally more polar, and so more bioavailable, than the parent compounds [96]. An increase in bioavailability of polar metabolites was demonstrated by experiments undertaken with the white rot fungus *Bjerkandera* strain BOS55 [97]. As a pure culture it was able to degrade 74% of ^14^C-benzo*a*pyrene but only produced a limited amount of ^14^CO_2_. The addition of soil, sludge or LMW PAH enrichment cultures led to a rapid increase in ^14^CO_2_ production as the polar metabolites produced by the fungus were mineralised, but only up to 34%, indicating that some ^14^C- benzo*a*pyrene fungal metabolites were readily biodegraded while others persisted [97]. This has also been demonstrated with fungal-bacterial co-cultures containing the non-ligninolytic fungus *Penicillium janthinelum* VUO 10,201, which showed significant degradation of a range of HMW PAHs including pyrene and benzo*a*pyrene compared with either the fungal or bacterial species incubated alone [98]. Twenty-five percent of benzo*a*pyrene was mineralised to CO_2_ over 49 days by the co-cultures, accompanied by the detection of transient intermediates [98].

**Figure 3:**
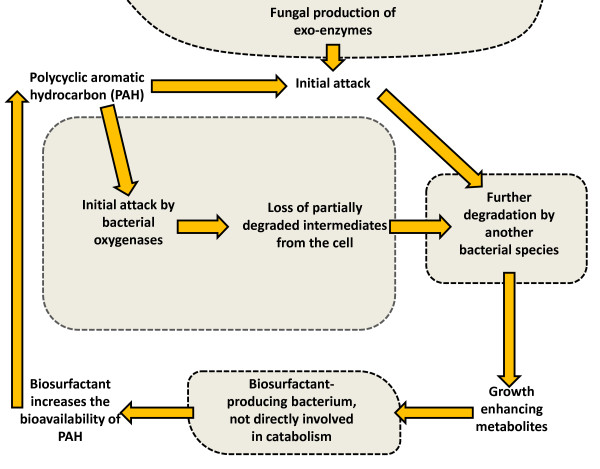
**Schematic illustration of some of the interactions seen in a microbial consortium degrading polycyclic aromatic hydrocarbons (PAHs).** Different microbial cells are represented by shaded shapes surrounded by a dashed line. Elements of these interactions have been seen in several studies (see text for details). Further complexity can be built into this simple schematic if one considers multiple PAHs invoking several pathways in one or more microbes, as well as co-metabolic degradation.

Figure 3 provides a schematic illustration of some of the interactions involved in hydrocarbon biodegradation. When present in mixtures, PAHs have the capacity to negatively influence the rate and extent of biodegradation of other components in the mixture [99]. Some metabolites may not be degraded further in a particular environment (dead-end metabolites), and while they are usually less toxic than the parent compound, some are more toxic, and so it is important to monitor production of metabolites and the overall toxicity during bioremediation processes. For example, metabolites, such as pyrene-4,5-dione derived from pyrene transformation have the potential to accumulate in PAH-contaminated systems and significantly inhibit the biodegradation of other PAHs [100]. 

Although fungi are considered to be largely terrestrial, they have been found in marine mats [101] and it is known that many can function in saline conditions [102], but in general salt-adapted fungi have received little attention despite a potentially major role in coastal PAH degradation. The ubiquitous co-existence of bacteria and fungi in soil and sediments [103] and their known catabolic cooperation suggests that physical interactions between them may be of importance for PAH degradation. There is also evidence that filamentous fungal networks may facilitate the movement of hydrocarbon-degrading bacteria through soils and sediments – the so-called “fungal highway” – by providing continuous liquid films in which gradients of chemo-attractants can form and chemotactic swimming can take place, thus greatly increasing the accessibility to pollutants [104].

## Biosurfactants and the interactions between hydrocarbon-degrading microbes and their environment

PAHs are usually found mixed with other organic pollutants (commonly petroleum and derived products) in contaminated sites, which may alter their fate and transport. This is of particular relevance when considering aged or weathered oils, in which PAHs will be less bioavailable because they are more effectively partitioned within the residual oil phase [105]. PAHs, particularly HMW PAHs, adsorb strongly to minerals and their associated organic matter [106], further diminishing their bioavailability. Owing to the low solubility and high levels of adsorption of PAHs, many microbes have evolved mechanisms to access them more readily. For example some PAH-degrading microbes have high-affinity uptake systems that efficiently reduce the PAH concentration close to the cell surface, thereby enhancing diffusive flux [107,108]. Living on the mineral surfaces to which PAHs are adsorbed is another strategy that reduces diffusion time [109] by physically reducing the distance between cells and substrate. During such interactions, the nature of the cell surface is extremely important; for example the mycolic acids of mycobacteria and related Actinobacteria enhance cell-surface hydrophobicity which serves to encourage biofilm formation and uptake of lipophilic compounds into the cell [110]. The production of extracellular polymeric substances (EPS) has also been shown to be an important mechanism in allowing attachment of *Pseudomonas putida* to solid PAHs [111]. Vaysse *et al*. [112] showed that *Marinobacter hydrocarbonoclasticus* exhibited a major change in the proteome of cells freshly detached from hexadecane compared with those attached to hexadecane. Their mobilization may be fuelled by intracellular wax esters accumulated while growing as a biofilm on hexadecane, and the dispersed cells demonstrated a high capacity to reattach to the *n*-alkane [112]. Thus, the ability to readily attach to hydrocarbons and then move to a new patch appears to be essential for many hydrocarbon-degrading bacteria. During this process the hydrocarbon surface will be modified by excreted microbial products, and would thus be expected to lead to colonization by a succession of different microbes, however we are not aware of any studies exploring this in detail. Wouters *et al*. [113] used differential fluorescence staining to analyse a model, three-species community on the surface of PAH crystals, which looks like a promising tool to investigate their interactions and succession on the hydrocarbon surface.

Another mechanism for increasing the bioavaliabilty of these compounds is the production of biosurfactants (biological surface-active agents that have both hydrophilic and hydrophobic moieties). Some biosurfactants are known to inhibit certain microbes, while at the same time benefiting others by increasing the bioavailability of hydrophobic compounds that can serve as a carbon and energy source, thus acting as “common goods” [79]. Numerous studies have shown that production of biosurfactants, by either degrading or non-degrading microbes, is essential in enhancing the bioavailability of poorly soluble and adsorbed hydrocarbons [114,115]. Low-molecular-weight biosurfactant molecules are mostly glycolipids, including rhamnolipid, trehalose lipids and sophorolipids, or lipopeptides such as surfactin, gramicidin S, and polymyxin [114,115]. High molecular weight EPS can also act as a biosurfactant, and represents a heterogeneous range of polymers composed of polysaccharides, proteins, lipopolysaccharides, lipoproteins or complex mixtures of these biopolymers [114,115]. Biosurfactants preferentially partition at the interface between polar and apolar molecules (e.g. hydrocarbons and water), producing micro-emulsions which in many cases enhance bioavailability and desorption of the hydrocarbon [115].

McKew *et al*. [30] demonstrated that the addition of *Alcanivorax borkumensis* to seawater microcosms containing crude oil, increased PAH-degradation rates despite the fact that *A. borkumensis* does not mineralise PAHs. *A. borkumensis* is known to produce biosurfactants, which enhance uptake of alkanes, its main source of carbon and energy [38]. It is probable that such biosurfactants produced by *A. borkumensis* fortuitously increase the availability of PAHs thereby enhancing their biodegradation by other microbes in the seawater [30]. The release of such “common goods” may benefit *A. borkumensis* by reducing the concentration of stress-inducing PAHs, however those PAH-degraders will be competing for nitrogen and phosphorous that are commonly limiting nutrients in petroleum-contaminated environments. Furthermore, the biosurfactants may benefit other alkane degraders competing directly with *A. borkumensis* for alkanes. *Rhodanobacter* strain BPC1 from an eight-strain consortium degrading benzo*a*pyrene in a mixture of diesel fuel components, was found to be the pivotal organism in making benzo*a*pyrene ~500 times more soluble, thus enhancing its degradation [116]. Strain BPC1 was unable to grow on the mixture, but grew in the presence of the other microbes, indicating that it was probably utilizing metabolites produced by other consortium members [116]. Similarly, the addition to seawater of EPS from *Rhodococcusrhodochrous*. S-2, that serves to protect this strain from aromatic-hydrocarbon-induced stress, enhanced crude oil degradation and stimulated the growth of *Alcanivorax* and especially *Cycloclasticus* spp. [117]. Although *Cycloclasticus* spp. grow in pure culture, they are frequently difficult to maintain, which together with the above observations [30,117], suggests that in nature they may typically take advantage of biosurfactants produced by other microbes.

Biosurfactants may also serve an antagonistic role – they are after all important virulence factors in many pathogens – and their effects will be dose- and species-dependent. Rhamnolipid generally enhances hydrocarbon bioavailability and degradation [30,118], but Shin *et al*. [119] reported that it inhibited degradation of phenanthrene by a two-species consortium of *Sphingomonas* and *Paenibacillus* sp., even though in pure culture the rhamnolipid inhibited only *Sphingomonas* sp. It was therefore suggested that the increased stress caused by the solubilized phenanthrene, or the rhamnolipid in the presence of solubilized phenanthrene, was responsible for inhibition of *Paenibacillus* sp. It is also important to consider the potential synergistic role of multiple biosurfactants. Rambeloarisoa *et al*. [120] studied an eight-strain microbial consortium from the French coast, and found that biosurfactants produced by a pure strain did not emulsify crude oil, whereas those produced by the whole bacterial community did emulsify oil and led to rapid hydrocarbon degradation [120]. The extent to which such multi-species synthesis of biosurfactants may be coordinated remains to be discovered. Microbial, petroleum and clay interactions are important but very poorly understood. Chaerun *et al*. [121], for example, showed that montmorillonite and kaolinite enhanced growth on heavy oil, acting as supports for microbes producing EPS, as well as buffering the pH. Degradation of adsorbed PAHs involves specific adaptations that are still not well understood, and some microbes specialise in accessing and degrading adsorbed PAHs [107]. Vacca *et al*. [122] showed that none of the 25 soil strains isolated with non-sorbed phenanthrene could mineralise humic-acid sorbed phenanthrene (HASP), whereas all three strains that were enriched on HASP were proficient at mineralising it, clearly indicating that different capacities are needed for the biodegradation of adsorbed PAHs.

## Microbial interactions during anaerobic degradation of hydrocarbons

Biodegradation of hydrocarbons in anoxic marine sediments is slower than in oxic zones, and it is generally assumed that the primary mechanism of hydrocarbon degradation even in marine sediments is aerobic respiration [123]. Despite the absence of oxygen to activate hydrocarbons, other mechanisms [124] can lead to the initiation of their degradation by a wide range of anaerobic species utilising diverse terminal electron acceptors [124]. In the environment, anaerobic hydrocarbon biodegradation is most likely to involve syntrophic consortia. Conversion of *n*-hexadecane to methane in an anaerobic enrichment culture was shown to involve a consortium of microorganisms, which on the basis of phylogenetic affiliation had the following putative phenotypes: syntrophs belonging to the Syntrophaceae (called *Syntrophus* but probably *Smithella*[125]) that convert *n*-hexadecane to acetate, hydrogen and CO_2_; methanogens that convert acetate to methane and CO_2_; other methanogens that convert hydrogen and CO_2_ to methane; and a *Desulfovibrio* sp. that may couple hydrogen and CO_2_ consumption with sulfate reduction [126]. However, a fermentative, syntrophic role for *Desulfovibrio* sp. must be considered given its metabolic flexibility [127]. A methanogenic consortium with a remarkably similar structure was also found to degrade toluene [128]. Such microbial teamwork is common in the anaerobic mineralisation of structurally complex compounds. The syntrophic association is important because the methanogens lower the concentrations of hydrogen and acetate, which makes the breakdown of the alkane energetically favourable. It will be important to elucidate the precise nature of such interactions involved in the thermodynamically challenging anaerobic degradation of hydrocarbons, particularly identifying the microbes responsible for the initial activation and their mode of action [129]. The extremely high level of enrichment in methanogenic hydrocarbon-degrading consortia provides strong evidence [125,126] that *Smithella* spp. play this role. Better means of identifying and tracking intermediate metabolites will also be essential to better understanding the mechanism of these closely coupled syntrophic consortia.

It is important to consider that in many environments a gradient of oxygen concentrations can be found, with consequent microbial adaptations to a microaerobic lifestyle. Benzene degradation, for example, has been shown to occur at 0.05 mg l^-1^ of oxygen [130]. Moreover, aerobes and anaerobes can co-exist in chemostats [131,132]. For example, the strict aerobe, *Comamonas testosteroni*, and strict anaerobe, *Methanosarcina barkeri*, grew together, with the aerobe consuming the oxygen and maintaining it at a sub-inhibitory concentration for the methanogen [132]. Similar mixed cultures were detected in a benzene-contaminated aquifer [133], but the nature of the interaction *in situ* remains to be elucidated. Diurnal fluctuation in photosynthetically derived oxygen is an important consideration in coastal biofilms, and sequential aerobic-anaerobic hydrocarbon degradation may be an important mechanism. For instance, Chayabutra and Ju [134] investigated the sequential degradation of *n*-hexadecane by *Pseudomonas aeruginosa* using aerobic resting cells in the initial aerobic mineralization and inducing nitrate-reducing conditions for subsequent anaerobic degradation of oxidized metabolites. Providing oxic-anoxic transitions for the treatment of oily sludge proved as effective as oxic conditions alone in the degradation of PAHs by a microbial community dominated by *Pseudomonas* spp. [135]. Rocchetti *et al*. [136] also compared microbial degradation of hydrocarbons under both oxic and anoxic conditions in addition to sequential oxic-anoxic treatment in microcosms containing contaminated sediments. They reported that hydrocarbon degradation was significantly enhanced via sequential anaerobic-aerobic degradation involving sulfate-reducing bacteria in the anaerobic step, compared to degradation under either aerobic or anaerobic conditions. A more thorough review of this topic that describes other outcomes as well as the effect of the starting conditions (oxic or anoxic) is provided by Cravo-Laureau *et al*. [137].

## Phototroph-heterotroph interactions

Marine phototrophs (primarily eukaryotic microalgae and cyanobacteria) contribute half the Earth’s primary production and half of the oxygen liberated to the atmosphere [138]. However, they do not exist in isolation, and their phycosphere (loosely defined as the zone around algal cells in which bacteria feed on algal products) constitutes an important habitat that is colonised by an abundant and diverse community of heterotrophic bacteria [72,139]. Bacteria are also found living inside microalgal cells - many with unknown function [140]. The composition of free-living marine microbial communities is frequently very different from those attached to microalgae [141], with certain groups often preferring the attached lifestyle [142] and showing higher levels of activity [143]. Moreover, different species of microalgae host distinct bacterial communities that change with time and environmental conditions [72,144]. However, there is likely to be a large spectrum of bacterial heterotroph-phototroph specificity [145], and certainly many attached bacteria can also live in the absence of a microalgal or cyanobacterial host [146]. While antagonistic interactions occur between marine phototrophs and their attached microbiota [147,148], mutualistic interactions are common, with the host supplying carbon and energy sources [149], as well as potential protection from desiccation and grazing via their EPS; while the bacteria have been shown to provide iron [150], haem [151], vitamin B12 [152], to consume oxygen [153] and provide protection from reactive oxygen species [154]. Symbiotic cyanobacteria supply fixed nitrogen to diatoms [155] and other algae and protists [156], and heterotrophic N_2_-fixing bacteria may also be important in interactions with microalgae, as evidenced by the abundance of alphaproteobacterial diazotrophs in seawater size fractions of >10 μm [157]. Attached bacteria can affect microalgal morphogenesis [158], the composition of their EPS [159] and enhance aggregate formation [160]. Indeed, many microalgae function less efficiently or do not even grow as axenic cultures [161]. Bruckner *et al*. [162] showed that a complex network of chemical cues, including amino acids and EPS, may be involved in regulation of diatom-bacteria biofilms. The variety of metabolites released from both microalgal and bacterial cells is immense [163], and dissecting out those that are important or essential for nurturing specific or general interactions is a major task for the marine biochemist.

Such heterotroph-phototroph interactions are of direct relevance to hydrocarbon degradation, not least because oil has most environmental impact where it floats on the sea surface and especially intertidal areas where microalgal biofilms are usually dominant. Although the water-soluble fraction from oil was shown to reduce the abundance of marine phytoplankton (primarily *Prochlorococcus*), the effect on coastal planktonic diatoms was stimulatory for small (<20 μm) species and either inhibitory or stimulatory depending on the concentration for larger diatoms [164]. Many marine phototrophs can withstand high concentrations of crude oil, and some cyanobacteria appear to accumulate hydrocarbons without degrading them in inter-thylakoid spaces [165]. Coastal biofilms are particularly resistant to oil pollution, which can even result in enhanced photosynthetic activity [166]. The cyanobacterial genus, *Oscillatoria*, is particularly common in oil-polluted mats [167-169]. Diatoms too are often abundant in diverse oil-polluted sediments, including a chronically oil-polluted lagoon in which diatom chloroplast 16S rRNA gene sequences constitute up to 21% of the sequences from the surface sediment [170].

Although there are many reports of hydrocarbon degradation directly by microalgal species, primarily chlorophytes and diatoms (as summarised by Prince [17]), but also cyanobacteria, it is questionable whether microalgae would be competitive with specialist aerobic heterotrophs, and they are probably involved only in partial oxidation [171-174]. For example, Todd *et al*. [174] showed that the chlorophyte, *Chlorella vulgaris* slowly metabolized naphthalene to 1- naphthol. However, other evidence implicates photo-(mixo)trophs in complete hydrocarbon oxidation. For example, fatty acid analysis of cyanobacteria grown with and without hydrocarbons, suggests that they are incorporated into biomass [175]. Also, Lei *et al*. [176] reported that six strains from diverse microalgal genera, including *ChlamydomonasChlorella, Scenedesmus, Selenastrum and Synechocystis*, could degrade 34 to 100% of the supplied pyrene in 7 days.

It is difficult to obtain axenic cultures of microalgae, and so in some reports of more complete and rapid hydrocarbon degradation by phototrophs the degradation could have been performed wholly or partly by associated microbes [177]. For example, the medium used to check for the absence of heterotrophic bacteria in cyanobacterial cultures that degraded 50% of hexadecane and up to 90% of PAHs in 10 days [178] contained peptone-glucose that would not have allowed *Alcanivorax* spp. to grow, and so they would evade detection. De Oteyza *et al*. [179] have shown that while cyanobacterial filaments surround oil droplets, biodegradation was most likely due to associated heterotrophic bacteria. Cohen [168] found rapid degradation in cyanobacterial mats, whereas pure cyanobacterial cultures could not degrade hydrocarbons. Therefore, while cyanobacteria-dominated mats can degrade hydrocarbons, it is the heterotrophic bacteria that are mainly responsible for the degradation [166,168,177,180-182]. However, it is important to determine the extent to which microalgal biodegradation of hydrocarbons and their metabolites [173,183] is relevant in the marine environment.

Phototroph-heterotroph interactions are very important to hydrocarbon biodegradation. Many algae produce hydrocarbons [184,185], and nearly all produce the volatile hydrocarbon, isoprene [186,187], which could serve to sustain hydrocarbon-degrading communities in the absence of an oil spill [188], and may explain why hydrocarbon-degrading bacteria, such as *Alcanivorax* spp., are often associated with micro-[189] and macro-[190] algae. PAHs adsorb to the cell surface of marine microalgae at relatively high concentrations [191], and have been shown to be transported by phytoplankton cells from the surface layers of the Southern Baltic to the sea floor [192]. Thus, exogenous hydrocarbons may also support hydrocarbonoclastic bacteria attached to algae. Other bacterial genera that have many species with the capacity for hydrocarbon degradation, such as *Marinobacter* and *Roseobacter*, are also commonly associated with algae [144,160,168,189,193]; however both are nutritionally versatile and so could use diverse sources of carbon and energy supplied by their hosts. Gutierrez *et al*. [59] isolated a new species of specialist PAH degrader, named *Porticoccus hydrocarbonoclasticus*, from the marine dinoflagellate *Lingulodinium polyedrum*, and also used quantitative PCR to show that it was associated with other phytoplankton.

**Figure 4:**
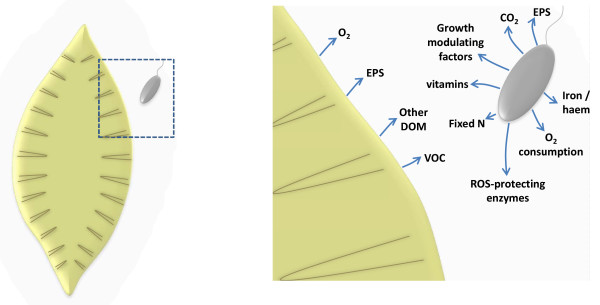
**Schematic illustration of the transfer of metabolites between a photoautotrophic alga (gold) and an organoheterotrophic bacterium (dark grey) embedded in algal extracellular polymeric substances (light grey).** The right-hand diagram is an expansion of the area in the box. EPS = extracellular polymeric substances, DOM = dissolved organic matter, VOC = volatile organic compounds, ROS = reactive oxygen species.

Figure 4 shows some of the means by which algae and associated bacteria collectively interact, as discussed previously. These associations may be enhanced by the presence of hydrocarbons; for example oxygen liberated by photosynthesis is likely to be very important in activating hydrocarbons and serving as an electron acceptor in aerobic respiration [75,168,179,194]. In turn, the locally increased concentration of CO_2_ produced by the heterotrophs, will generally allow enhanced photosynthesis. Abed [194] studied the interactions between cultivated cyanobacteria and aerobic heterotrophic bacteria in the degradation of hydrocarbons, showing an increase in growth of the bacteria and enhanced hydrocarbon degradation in the presence of cyanobacterial organic exudates. Similarly, extracts from a chlorophyte enhanced benzo*a*pyrene degradation by a *Mycobacterium* sp. and *Sphingomonas* sp. [195]. The consortium constructed by Tang *et al*. [196] consisted of an alga, *Scenedesmus obliquus* GH2, that could not degrade petroleum hydrocarbons but promoted the degradation of both aliphatic and aromatic hydrocarbons (especially HMW PAHs) by the added bacterial members of the consortium. In this interesting study it was also observed that when a unialgal, but non-axenic, culture of *Scenedesmus obliquus* GH2 was added to the consortium, degradation was inhibited, implying that unidentified non-hydrocarbon-degrading bacteria associated with the alga outcompeted the added oil-degrading bacteria. 

The organic compounds produced by algae may influence hydrocarbon degradation in different ways. Algal EPS could serve to emulsify hydrocarbons as suggested by Cohen [168]. Additionally, EPS together with excreted amino acids and sugars provide a source of carbon and energy for associated bacteria (as well as the microbial community beyond the phycosphere) [197]. It is not known what effect algal dissolved organic matter (DOM) might have on hydrocarbon biodegradation, but in other environments the addition of organic compounds led to both increased and decreased hydrocarbon consumption [198-200]. Such simple organic compounds significantly enhance microbial populations, a proportion of which may also have the capacity to degrade hydrocarbons. (This is the explanation often given for the success of phytoremediation of polluted land, where plant-root exudates stimulate microbial growth). Alternatively, the stimulated populations may out-compete hydrocarbon-degrading bacteria, especially obligate hydrocarbonoclastic species. In summary, we do not yet have a mechanistic explanation for the above [194-196] observations of stimulation of hydrocarbon degradation by algal exudates. The possibility should also be considered that algae produce secondary metabolites to specifically nurture hydrocarbonoclastic bacteria, as removal of stressful hydrocarbons would benefit the host.

Hydrocarbon-degrading bacteria could supply the algae with the benefits outlined in Figure 4. *Alcanivorax* and *Marinobacter* spp., for example, are well adapted to sequestering iron [40,150]. Most importantly, hydrocarbonoclastic bacteria will decrease the concentration and toxicity of hydrocarbons in the immediate vicinity of algal cells. There are several studies that demonstrate the benefit of such a co-culture; for example Abed [194] showed that the cyanobacterium *Synechocystis* sp. grew best in the presence of aerobic hydrocarbon-degrading bacteria and hexadecane. *Alcanivorax* spp., which have been shown to inhabit the phycosphere of algae such as the dinoflagellate *Gymnodinium catenatum*[189], can reduce the lag phase and enhance the maximum chlorophyll fluorescence of the cyanobacterium *Prochlorococcus* by means of diffusible molecules [201].

Nitrogen often becomes limiting in petroleum-contaminated environments [202], yet there are few studies on the impact of hydrocarbons on fixation of atmospheric nitrogen and in turn how this may influence biodegradation. Oil had little effect on nitrogen fixation in Arctic marine sediments [203] and marine-sediment microcosms [204], and had variable impact in salt-marsh sediments [205]. However, nitrogen limitation in other oil-polluted habitats can be overcome by dinitrogen fixation [206,207]. Musat *et al*. [204] demonstrated that cyanobacteria were the most active dinitrogen fixers in nitrogen-limited pristine and oil-polluted marine sediments reconstructed in aquaria, by combining acetylene-reduction assays with light–dark incubations and sequence analysis of expressed *nif*H genes. The capacity to fix atmospheric nitrogen and solubilise phosphate should be advantageous for microbes that rely largely on a diet of hydrocarbons. Also, the ability to scavenge iron, a major component of hydrocarbon-activating oxygenases, would be important in oligotrophic environments. There was little data suggesting that these capabilities may be widespread in hydrocarbon degraders until two recent studies showed that many hydrocarbon-degrading bacterial isolates potentially [208] or actually [190] fix nitrogen, and 84% of isolates produced siderophores to access iron and 51% solubilised phosphate [208].

## Grazers and viruses

In order to better understand natural attenuation and determine the potential for bioaugmentation of oil-contaminated marine environments, it is essential to understand the effect of oil on grazers [27]. Grazing organisms play a role in the transfer of hydrocarbons or their metabolites to higher trophic levels, and also affect degradation rates, both positively and negatively [209]. It is pertinent to ask whether hydrocarbonoclastic bacteria forming biofilms on oil droplets are grazed by protozoa (e.g. ciliates and flagellates) or meiofauna (e.g. nematodes, copepods and ostracods) to the same extent as other bacteria. The grazer would have to avoid co-ingestion of oil or subsequently tolerate or expel it. Stoeck and Edgcomb [209], summarising the rather scant literature on this topic, state that defence mechanisms include release of protective mucous and complexation of hydrocarbons with lipids. Many grazers are resistant to crude-oil components, for example Gertler *et al*. [210] found an abundant, fluctuating protozoal community alongside an abundant, inversely fluctuating and active hydrocarbon-degrading bacterial community in a marine mesocosm. The main protozoal species changed over time, with selection in the oiled mesocosm of *Scuticocilitia* spp. initially and *Euplotes* spp. later, both of which had been found by other researchers in polluted environments [210]. Also, Dalby *et al*. [211] concluded that cosmopolitan generalist protozoa could effectively graze bacteria in crude-oil amended microcosms. In the presence of oil, the flagellate, *Paraphysomonas foraminifera*, became dominant (48-82% of 18S rRNA phylotypes), keeping the bacterial population below 10^7^ cells ml^-1^.

Grazing frequently leads to enhanced rates of organic matter mineralisation by releasing nutrients and/or maintaining heterotrophic populations in exponential growth phase [209]. However, there are few studies investigating the effects on hydrocarbon mineralisation, and the outcomes are sometimes conflicting, perhaps as a consequence of environmental differences or technical approaches. Using eukaryote inhibitors, Tso and Taghon [212] showed that grazing had a beneficial effect on naphthalene degradation in estuarine sediments, possibly because the protozoa selectively grazed those bacteria that were not attached to naphthalene, thus allowing attached naphthalene-degrading bacteria to flourish by reducing competition for nutrients and other resources. Mattison and Harayama [213] reported a four-fold increase in toluene mineralization by a *Pseudomonas* sp. in the presence of the bacterivorous flagellate *Heteromita globosa* than in its absence, though *Pseudomonas* numbers reduced to 60% of the original biomass in the presence of the flagellate. In this case it was suggested that, in addition to selectively grazing the less-active bacteria, *H. globosa* enhanced naphthalene degradation by excreting growth-stimulating metabolites or ammonium and phosphate. Rogerson and Berger [214] proposed that stimulation of crude-oil degradation by *Colpidium colpoda* may additionally have been due to increasing oxygen flow caused by the swimming action of the ciliate and/or production of oil-emulsifying mucus that may have enhanced hydrocarbon bioavailability. Stoeck and Edgcomb [209] provide examples of other indirect benefits of protozoa to oil biodegradation. In contrast, Näslund *et al*. [215] found that meiofaunal grazers reduced naphthalene degradation in marine sediments. By reducing the number of larger grazers, oil pollution can result in microalgal blooms [216,217]. Although the benefits of phototrophs have been outlined earlier, such a bloom may be disadvantageous because of algal competition for nutrients with hydrocarbon-degrading bacteria. More systematic studies investigating the role of different types of grazers under defined scenarios with varying levels of complexity are required to provide a clearer understanding of the nature of the interactions involved and the impact of grazers on hydrocarbon degradation.

*Bacteriovorax* spp. are obligate predatory bacteria that prey on other bacteria, but information regarding their potential role in oil-degrading communities is limited and conflicting. During hydrocarbon-degradation mesocosm experiments, *Bacteriovorax* were detected in microbial communities between days 21 and 35 [218] and days 21 and 28 [210]. However, in a similar experiment *Bacteriovorax* represented 11% of the bacterial community at day 0, but by day 15 none were detected [219].

Bacteriophages might also affect microbial oil degradation either positively or negatively. Pollutants can induce prophage [27,220], and the resultant bacteriophage-induced lysis of bacterial cells, unlike grazing, releases all cellular components back into the marine environment for reuse by other microbes. Such a phage-driven microbial-loop was implicated in enhancing total organic carbon removal in reactors treating oil-contaminated waters [221]. Rosenberg *et al*. [221] found extremely high densities of bacteria and phages in these reactors, and they isolated phages, including one that infected a strain of *Marinobacter* cultured from the same location. Using the GeoChip-based high-throughput microarray, Lu *et al*. [222] observed significantly higher numbers of bacteriophage replication genes in the Deepwater Horizon deep-sea oil plume samples than in non-plume control samples collected at the same depth. Because previous studies had reported a significant increase in biomass in the plume samples [223], it was surmised that the bacteriophages provided a constant supply of nutrients needed for bacterial hydrocarbon degradation through phage-mediated biomass turnover. Furthermore, phages, together with various mobile genetic elements, are important in dissemination of valuable genetic material, including hydrocarbon-degradation genes and in the generation of new catabolic pathways via lateral gene transfer [224,225].

## A brief overview of microbial interactions with macrofauna and plants

There exists substantial evidence that bioturbation by larger fauna has a significant impact on the degradation of petroleum hydrocarbons in oil-contaminated sediments. By selective-removal experiments, Cuny *et al*. [226] found that the marine polychaete, *Nereis diversicolor*, increased the abundance of bacteria known to play important roles in aerobic hydrocarbon degradation. It was suggested that digestive solubilizers produced by the polychaete via feeding might have enhanced the bioavailability of the hydrocarbons and/or burrowing activities enhanced oxygen transfer to hydrocarbon-degrading bacteria. Gilbert *et al*. [227] had demonstrated previously that the digestive process of the polychaete *Nereis virens* altered the composition and reduced the concentration of ingested aliphatic hydrocarbons. It was therefore surmised that surfactant production in the gut of the worm led to these changes in the hydrocarbons. In addition to aerating deeper sediments, burrowing animals may transport pollutants or degrading bacteria deeper into sediments or return buried pollutants back to the surface [228,229].

Plant roots oxygenate their rhizosphere and provide sugars and other compounds that stimulate microbial activity; and ultimately their major polymers, such as lignin, upon entering the soil will be attacked by a suite of (fungal) extracellular enzymes, which will initiate fungal degradation of PAHs. Phytoremediation, which exploits these features, has been employed in terrestrial soils, but only trials have been carried out in coastal zones [230]. For example, Lin and Mendelssohn [231] investigated both tolerance limit to crude oil and phytoremediation potentials of the salt-marsh grass *Spartina patens*. It could survive at concentrations up to 320 mg oil g^-1^ dry sediment, and at oil doses of between 40 and 160 mg g^-1^ oil degradation was significantly higher than in unplanted sediments. The rhizopheres of mangrove species were shown to harbour a variety of bacteria that both degraded oil and potentially stimulated plant growth [208]. As with algal-bacterial interactions, a more complete understanding of the molecular interactions between plants and associated bacteria and fungi will only improve the possibility of this technology being rationally applied to remove oil in the coastal zone [232].

## Concluding remarks and prospects for using interacting microbes for oil-spill cleanup

There has been a lot of debate about the validity of bioaugmentation, specifically supplementing the environment with microbes to enhance biodegradation or detoxification of pollutants. Examples of success and failure abound. The key reasons for failure include: use of a single organism, focus on biodegrading strains only, microbes not adapted to the environment, inadequate dispersion/ access to the pollutant, lack of protection (e.g. from grazers), other factors limiting biodegradation (e.g. nutrients). Now, there is overwhelming evidence that using a consortium of microbes rather than a single strain greatly enhances the chances of successful bioaugmentation.

A well designed microbial consortium will have complementary catabolic pathways, as well as the potential to disperse and make the hydrocarbons readily bioavailable. Gallego *et al*. [233], for example, demonstrated the vastly superior efficacy of a designed four-species consortium over individual species in the bioremediation of oil-tank sludge. A six-species manufactured consortium, including a fungus, *Fusarium* sp., mineralised 78% of the PAHs from soil in 70 days, compared with negligible mineralization in an uninoculated control, and much lower degradation with single-species inocula [234]. Successful bioaugmentation is also a function of the competition between the introduced microorganisms and the autochthonous microbial community, and the study of this biotic pressure requires more attention.

Despite the improved biodegradation of hydrocarbons in bacterial co-cultures with microalgae, there have been few attempts to exploit this in the remediation of petroleum contamination. Munoz and Guieysse [235] describe *ex-situ* bioremediation using photobioreactors, but for marine pollution an *in-situ* approach is preferred owing to the large volume of polluted material. The critical phase of crude-oil contamination of the shoreline is the first few days. If the oil is not rapidly degraded then it will start to sink into the sediment where it can remain for decades. While it is true that hydrocarbonoclastic microbes will emerge from the native community, this process may take days. Thus, there is a role for bioaugmentation to bolster the *in-situ* hydrocarbon-degrading community in this crucial period. The potential to apply relevant hydrocarbonoclastic bacteria with or without associated microalgae should be investigated further.

Clearly there are many fundamental gaps in our understanding of microbial interactions; however, by a combination of reductionist experiments through to modelling the co-occurrence of microbial communities on a large scale, the field is advancing. The nature of interactions can be captured by single-cell and *in-situ*-metabolism imaging techniques such as Raman-FISH [236] and Nano-SIMS [237], as well as co-localisation studies using Magneto-FISH [238]. The requisite tools are constantly being developed, such that we can characterise and analyse in more depth the function of diverse components of DOM or the epimetabolome, as well as the volatile organic compounds, including the all-important signalling molecules. It is essential to make greater sense of metabolomics and protein and gene expression analyses in microbial consortia via the tools of systems biology [86,239]. A better understanding of microbial community metabolic networks will arise from recreating natural consortia in which modifications can be made a gene at a time. The result will be a clearer picture of microbial interactions and thus the functioning of global biogeochemical cycles, with potential practical offshoots, not least a more rational approach to the remediation of marine pollution.

## Competing interests

The authors declare that they have no competing interests.

## Authors’ contributions

TJM conceived the review and wrote the first draft. All authors contributed to the writing and read and approved the final manuscript.
